# Sine cosine optimization algorithm combined with balloon effect for adaptive position control of a cart forced by an armature-controlled DC motor

**DOI:** 10.1371/journal.pone.0300645

**Published:** 2024-05-16

**Authors:** Mohamed Tarek Mohamed, Salem Alkhalaf, Tomonobu Senjyu, Tarek Hassan Mohamed, Ahmed Mohamed Elnoby, Ashraf Hemeida

**Affiliations:** 1 Faculty of Energy Engineering, Department of Electrical Engineering, Aswan University, Aswan, Egypt; 2 Department of Computer, College of Science and Arts in Ar Rass, Qassim University, Ar Rass, Saudi Arabia; 3 Faculty of Engineering, Department of Electrical and Electronics Engineering, University of the Ryukyus, Nishihara, Japan; Sri Eshwar College of Engineering, INDIA

## Abstract

For a car that is propelled by an armature-controlled DC motor This study proposes an adjustable linear positioning control. In this paper, to optimize the parameters of the car’s position controller the sine cosine optimization algorithm (SCA) is utilized, with support from the Balloon effect (BE), The BE is incorporated to enhance the responsiveness of the traditional sine cosine optimization algorithm when faced with external disturbances and variations in system parameters. In the proposed approach, the determined value of the open loop transfer function of the motor and the updated values of the controller gains serve as the basis for the modified sine cosine algorithm’s objective function (OF). Under the influence of changes in motor parameters and step load disturbances, the system using the suggested controller is evaluated. Results from simulations and experiments show that the proposed adaptive controller, which implements the modified sine cosine algorithm, enhances the system’s overall performance in the presence of load disturbances and parameter uncertainties.

## 1. Introduction

### 1.1 Literature review

Although the DC motor has many benefits and uses in a variety of sectors, it also has certain disadvantages, including limited speed control, brushes and commutation, big size, electromagnetic interference, and expensive cost [1]. The DC motor is used in a variety of commercial applications, including those for robots, electric vehicles, the aerospace industry, industrial automation, energy production, medical sector and agriculture [2]. To control the DC motor, several controllers are proposed, different controllers have been showed in literature [3–6]. Amongst them, the (PID) controller is frequently used in industry Due to its efficiency and simplicity, improving both steady-state and transient performance. PID controllers have been used in many applications [7–9], including the DC motors control. However, it was necessary to develop online tuning techniques for the PID controller. In recent literature, there has been a surge in the adoption of optimization methodologies to fine-tune control parameters due to their effectiveness in mitigating uncertainties and disturbances [10–15]. Various optimization techniques have been explored in research papers, including a modified inverse neural controller [10], the Crow Search Algorithm [11], bee-colony optimization [12], particle swarm optimization (PSO) [13], Henry gas solubility optimization [14], and the grey wolf optimizer [15]. Furthermore, optimization techniques have been implemented for offline tuning of classic PI-LFCs as evidenced by studies such as [16,17]. These endeavors highlight the wide range of optimization approaches being employed to enhance controller performance and address control system challenges. since fixed parameters controllers created using nominal operating points are not suited for real-time operating situations. [18]. Several optimization algorithms, such as atom search optimization (ASO) [19], Zeigler-Nich reinforcement learning [20], and enhanced Bacterial Foraging Optimization (BFO) [21,22], have been proposed to compute finest parameters for the PID controller, Reinforcement learning: In this approach, the controller gains are adjusted based on the performance feedback obtained through trial and error. The controller learns from its own actions and continuously improves its performance. One of the popular reinforcements learning algorithms used for PID tuning is the Q-learning algorithm [23]. Additionally, techniques like fuzzy logic [24], genetic algorithms [25], and neural networks [26] have also been used for online tuning of PID parameters in various applications Model-based online tuning Using a model of the process dynamics, the controller gains can be adjusted in real time based on the current process state and desired performance criteria. This can be achieved using techniques like adaptive control or model predictive control (MPC) [27] a nature-inspired optimization technique that mimics the behavior of the sine and cosine functions called Sine Cosine algorithm (SCO) to solve optimization problems. This algorithm is widely used in various fields [28]. It has been successfully applied in Optimal power flow in electrical power systems [29], Parameter tuning in machine learning (ELDPs) [18], Routing optimization in wireless sensor networks [30], Energy-efficient scheduling in cloud computing [31] and Optimization of machining parameters. In the field of electrical DC motors. Furthermore, in order to enhance the effectiveness of optimization algorithms for dealing with system problems including load disruption and parameter uncertainties, a modification known as the balloon effect (BE) has been proposed [32].

### 1.2 Problem analysis

The adaptive control problem is solved using several optimization approaches in different ways. These methods may be used to modify the settings of neural network or fuzzy controllers [26], where the controlled variable’s error value serves as the basis for the objective function. There have been several efforts to directly optimize the adaptive controller’s parameters using optimization techniques. [27]. In these attempts, the objective function is constructed using time response characteristics such as overshoot, rise time, and settling time However, these methods have a drawback as they are based on nominal values of system parameters, thus making them less effective for time variant systems. To address this issue, a modification called BE has been proposed [24,25]. The objective function of the BE modification takes into account the new values of parameter variations and other system modifications. This allows standard optimization algorithms to be applied in adjusting control systems in real-time and industrial applications, such as load frequency control and motor control.

### 1.3 Contribution

The main contributions of this work can be summarized as:

- Proposing an adaptive position control method that utilizes sine cosine optimization technique with the support of BE for an industrial application represented by a cart forced by armature controlled DC motor and the proposed controller succeed to increase the precision of car desired position tracking.
- both digital/experimental tests assured how strong is the proposed adaptive control based SCO+BE in industrial applications.
- the suggested adaptive controller using SCO+BE has been used to make online tuning of load frequency controller in an isolated MGs and it proved efficiency in LFC issue in case of system difficulties such as load disturbance
- Demonstrating the effective problem-solving capabilities of the suggested control method by comparing the suggested optimization algorithm with other optimization techniques.
- To the best of the writers’ knowledge, this is the first time of utilizing SCO+BE for tunning the controller gains of the studded systems.

This manuscript is organized as the following; Section 2 discusses the sine cosine algorithm (Mathematical representation of classical SCO is presented in subsection 2.1 and Sine cosine algorithm combined with Balloon Effect (SCO+BE) is discussed in subsection 2.2) Section 3 designates the SCO+BE for position control of armature controlled DC motor. The SCO+BE for LFC of isolated microgrid is presented in Section 4. Section 5 discusses the conclusion. Section 6 suggests the future work.

## 2. Sine cosine algorithm

### 2.1 Mathematical representation of classical SCO

Researchers and practitioners in the field of optimization algorithms are continually looking for efficient and effective methods to address challenging optimization problems. Sine Cosine Optimization (SCO) is one such method that has received a lot of interest. By mimicking the natural oscillations seen in sine and cosine functions, which are mathematical ideas, SCO offers a novel method of optimization. Initially, a population-based metaheuristic optimization method was suggested for the SCO algorithm. It takes inspiration from sine and cosine waves, whose smooth motion displays periodic oscillations with predictable behavior. By simulating this natural occurrence, SCO hopes to quickly explore complicated solution spaces by striking a balance between exploration and exploitation during the search process [28].

**Fig 1:**
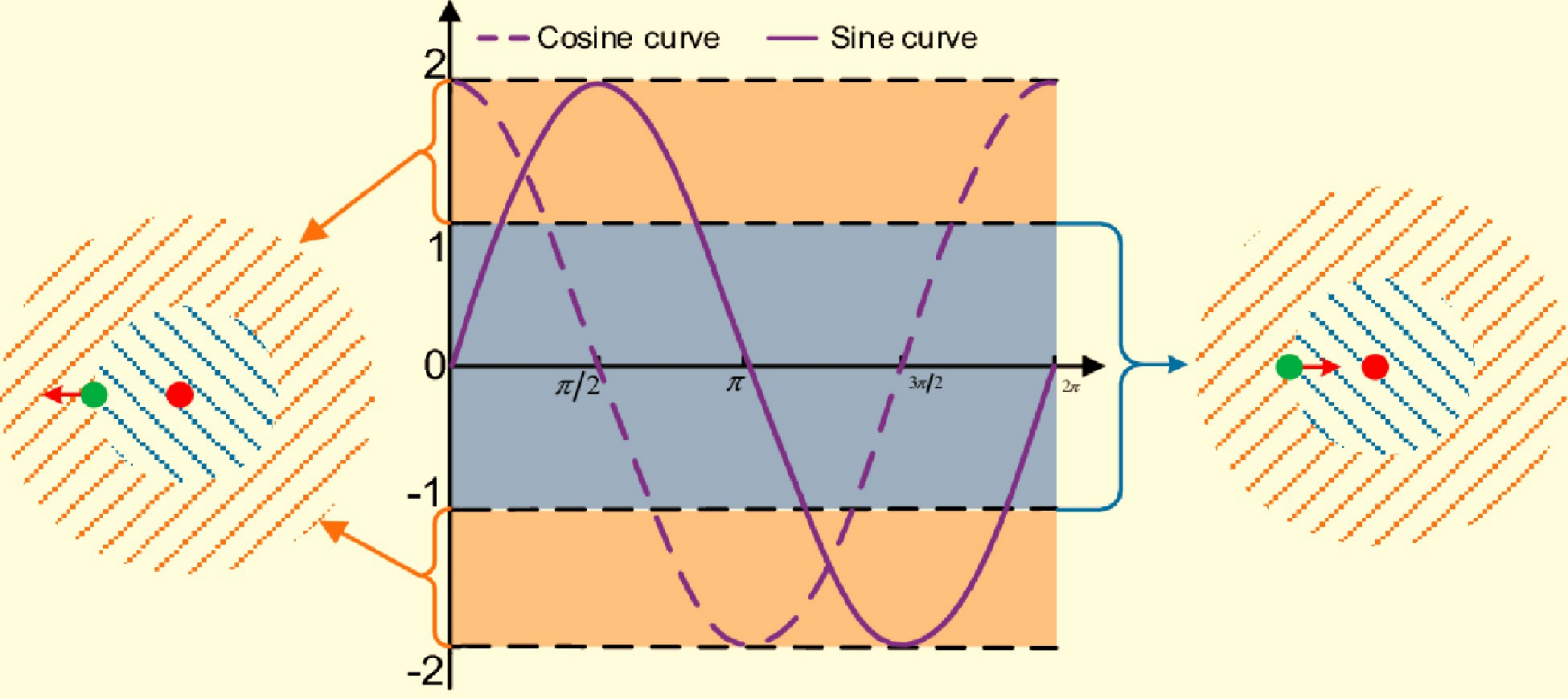
Diagram of SCA search mode.

The main idea behind SCO is that it can efficiently explore the search area and produce a variety of solutions. A population of potential solutions, each represented by a set of variables, is kept in memory by the algorithm. The direction and size of the movement for each unique solution are determined by the sine and cosine functions, which are used to update these variables [20]. Fig 1 represents Diagram of SCA search mode. SCO promotes exploration of unexplored areas while gradually convergent towards desirable portions of the solution space by combining these oscillatory dynamics with randomization. To guarantee the quality and diversity of the population, SCO uses a variety of components. These include adaptive parameters that regulate the trade-off between exploration and exploitation, individual best and global best solutions, and random initialization. Through iterative updates and interactions among the individuals in the population, SCO strives to find optimal or near-optimal solutions for a given optimization problem.

SCO is distinguished by its simplicity and ease of use in implementation. The method is known for its competitive performance across a variety of optimization issues and requires minimum parameter adjustment. Engineering design, image processing, banking, and machine learning are just a few of the areas in which SCO has been effectively used, exhibiting its adaptability and efficiency.

the SCO offers a promising approach to solving complex optimization problems. By drawing inspiration from the oscillatory behavior of sine and cosine functions, SCO strikes a balance between exploration and exploitation, enabling efficient search and convergence. With its simplicity, versatility, and competitive performance, SCO has emerged as a valuable optimization tool for researchers and practitioners alike [29,30].

(SCO) algorithm is a metaheuristic optimization algorithm that is used to solve optimization problems by iteratively adjusting a set of candidate solutions to find the optimal solution The first step in SCO is the initialization by Defining the problem’s search space boundaries and the number of iterations and initialize the population of candidate solutions with random values within the search space Then it calculates the Objective Function(of) for every solution and update each candidate solution using Eq (1)

Newsolution=currentsolution+r*(bestsolution‐currentsolution) (1)

where (r) is a random number between (0 and 1), then it Apply sine and cosine transformations to further update the candidate solutions for each variable in the solution, by using the Eq (2) and calculate the fitness of each updated solution

Newvariable=variable+A*sin(B*2*π*k)*|C*bestsolutionvariable‐variable| (2)

where A, B, and C are random parameters, and k is the current iteration number, then the algorithm Update the best solution by Comparing the fitness of the updated solutions with the fitness of the best solution found so far and if an updated solution has a better fitness, update the best solution finally the algorithm stops by reaching the maximum number of iterations or satisfies the convergence criterion.

The following information provides some notes about the Sine Cosine Optimization (SCO) algorithm:

- In SCO, a matrix with a number of items equal to the population size times the number of design variables represents the initial population. The best and worst values may be calculated using new solution values due to this matrix.
- for the same design variable in SCO, the initial values should be chosen close together to minimize sudden switches at the beginning of the optimization process.
- using a large population size and number of iterations can cause the SCO algorithm to run slowly, leading to poor real-time characteristics. To overcome this issue, it is recommended to set limitations on the number of iterations that may not necessarily result in the best solution but can yield better results than the standard algorithm.
- SCO can provide better and faster solutions by saving the ideal solution for next iterations.

**Fig 2:**
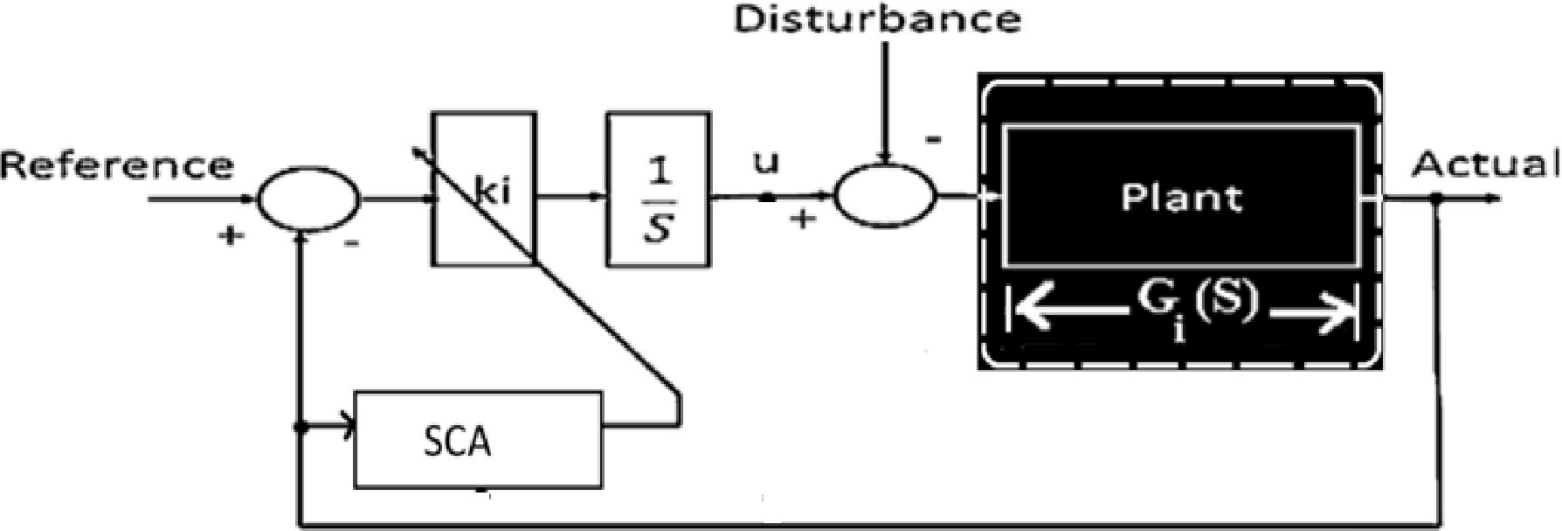
Traditional SCO system with open loop representation.

SCO is sometimes used in the issue of adaptive control to optimize the control inputs of a robotic manipulator. The algorithm was applied to tune the controller parameters based on the error between the desired and actual positions of the robot. This helped in improving the control performance and ensuring precise and smooth trajectory tracking. [26], and other times in controlling the unmanned aerial vehicles (UAVs), the SCO algorithm was utilized to optimize the UAV’s flight path and control parameters. By minimizing the cost function associated with trajectory tracking and energy consumption, the algorithm provided an efficient solution for adaptive control of UAVs in dynamic and uncertain environments. [27] Fig 2 shows an example of SCO being applied directly to optimize controller gains. The present case shows that, the rising time (Tr), maximum overshoot (Mp) and settling time (Ts) of the closed-loop system serve as the foundation for the objective function of SCO. These parameters are determined by the damping ratio (η) and natural frequency (ωn), which are elements of the theoretical open loop transfer function Go(S).

The classical SCO approach lacks the ability to adapt to variable disturbances or changes in system parameters in real-time. This limitation is a flaw in the traditional SCO’s application to the adaptive control problem.

### 2.2 Sine cosine algorithm combined with Balloon Effect (SCO+BE)

**Fig 3:**
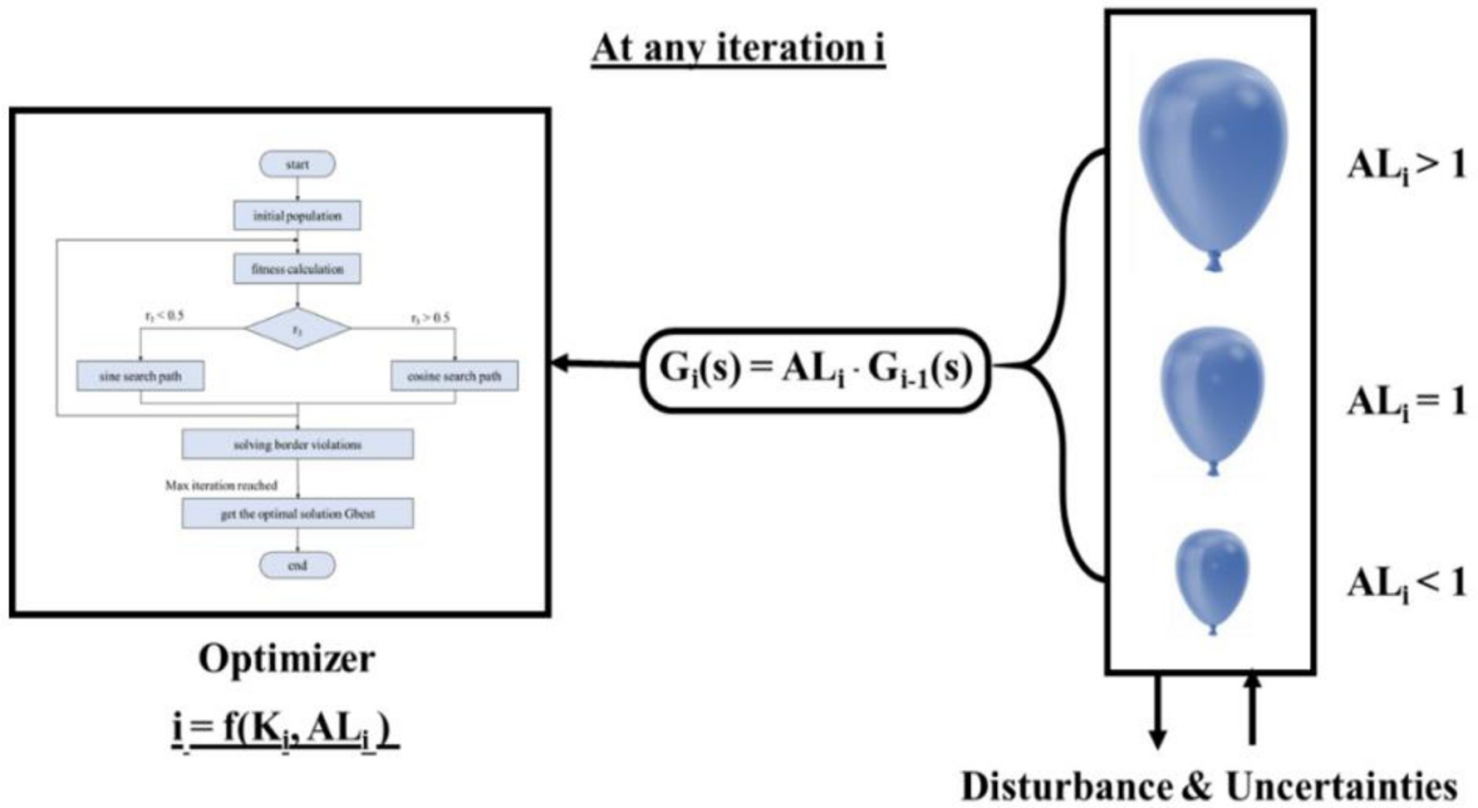
Idea of SCO with BE.

**Fig 4:**
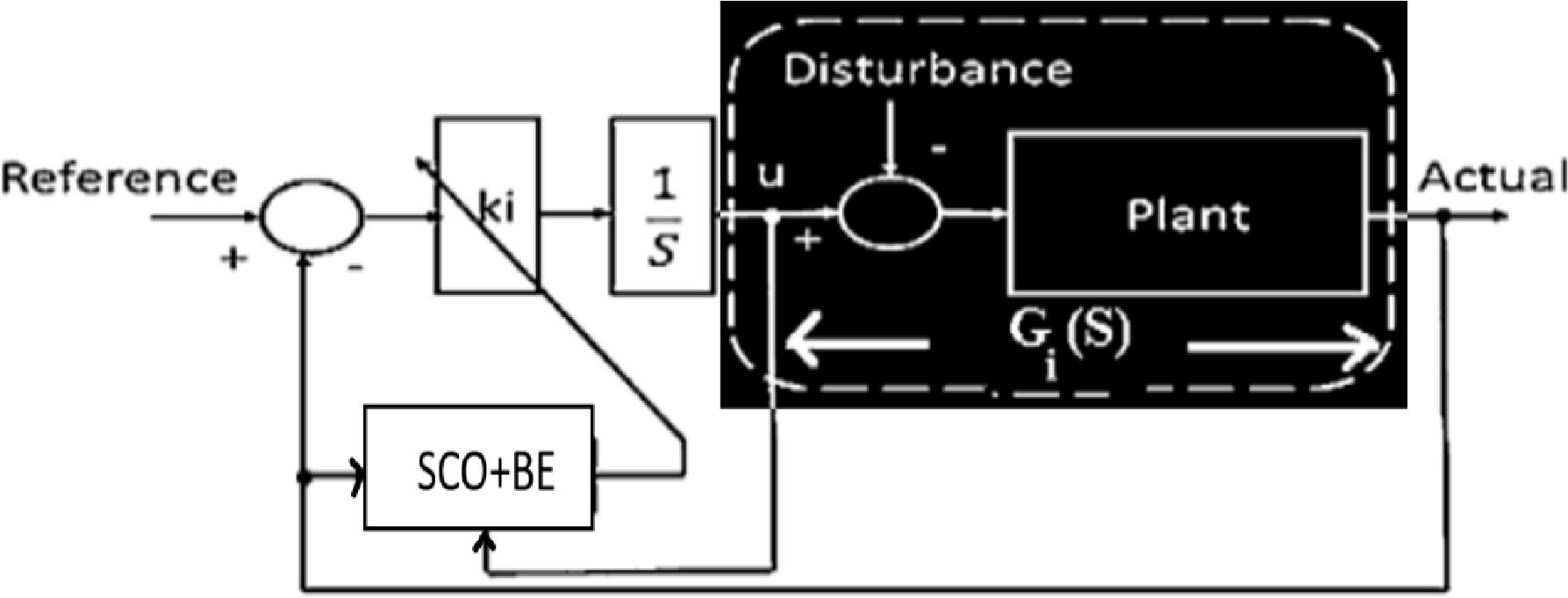
System with considering open loop representation for SCO with BE.

A feature called BE has been incorporated into the SCO algorithm in order to improve its efficiency in handling external disturbances or changes in plant parameters. This modified SCO aims to enhance its ability to respond to such disturbances or changes that may occur during any iteration. The concept of BE is illustrated in Fig 3, and the open loop transfer function utilized for the modified SCO at a certain time (i) is represented in Fig 4. The modified SCO approach can be summarized as follows:

1. The plant input *U*_*i*_and plant output *Y*_*i*_ are used to apply SCO during each iteration
2. By utilizing *U*_*i*_ and *Y*_*i*_, it is possible to compute the on-time transfer function.
$$G_{i}(S)=\frac{Y_{i}}{U_{i}}$$ (3)
3. The relation between *G*_*i*_(S) and *G*_*i*−1_(S)
$$G_{i}(S)=A{L_{i}}^{*}G_{i-1}(S)$$ (4)

At iteration (i) the impact of disturbance and changes in system parameters is represented by *AL*_*i*_. The shape of *G*_*i*_(S) changes depending on the value of *AL*_*i*_, similar to how a balloon shrinks and stretches affected by air pressure (as shown in Fig 2).

The relationship between *G*_*i*_(S) and *G*_*O*_(S) stated as:

$$G_{i}(S)=A{L_{i}}^{*}G_{i-1}(S)=(\prod_{n=1}^{i}AL_{n}{)}^{*}G_{o}(S)$$ (5)

4. The relation between *G*_*i*_(S) and *G*_*O*_(S) can be expressed using Eq (5). *G*_*i*_(S) depends on both *G*_*O*_(S) and $\left(\prod_{n=1}^{i}AL_{n}\right)$, which illustrates the presence of system problems. As a result, the SCO’ OF will change based on the values of system disruption and uncertainty levels, ultimately improving the performance of SCO.

**Fig 5:**
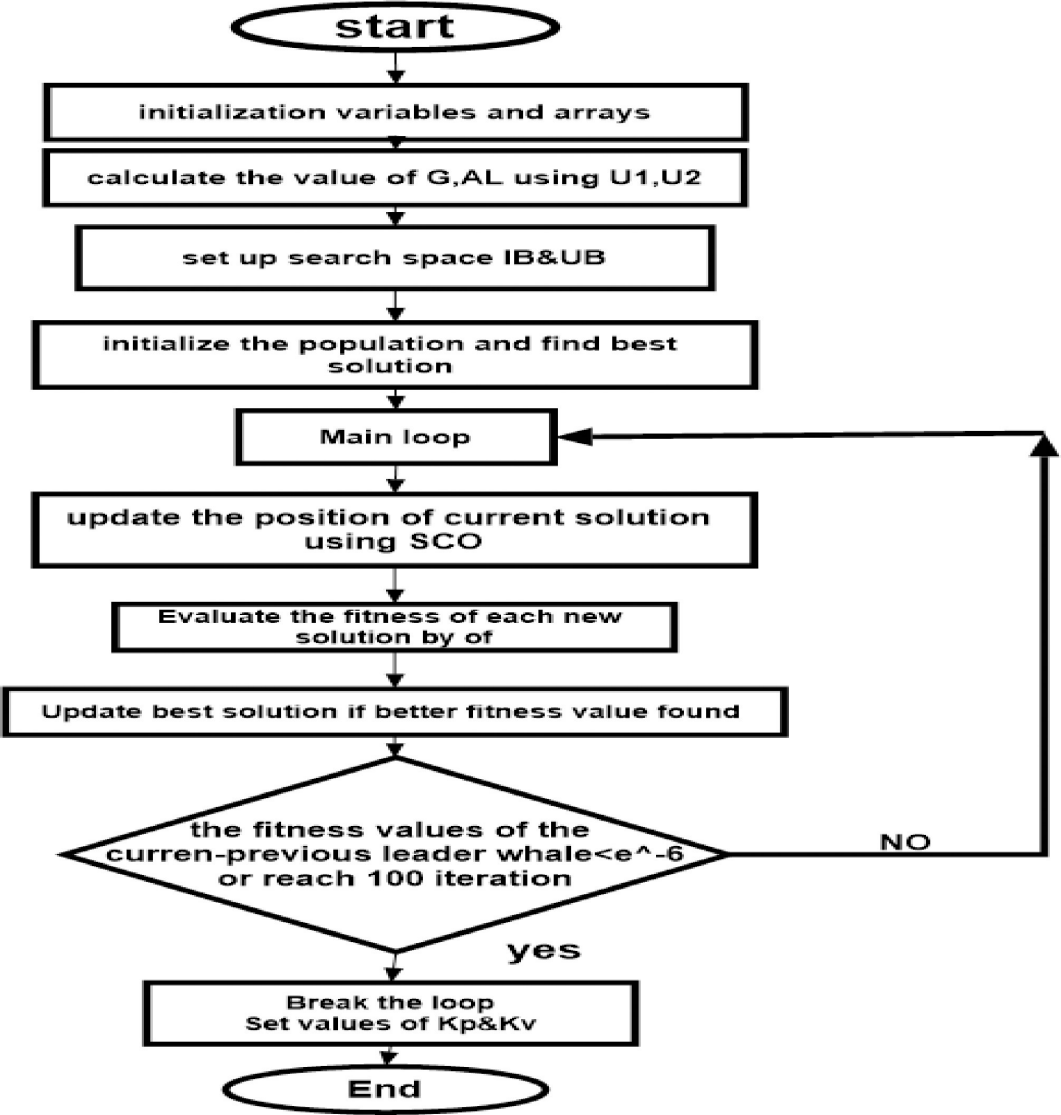
Flowchart represents SCO+BE.

Fig 4 illustrates the use of SCO + BE. Briefly, BE makes the objective function responsive to system variations at any given moment, increasing the efficiency of adaptive control optimized by SCO + BE. This makes it suitable for applications for industry such as load frequency control, thermal controland machine control. The flowchart of SCO with BE is shown in Fig 5.

a step by step of the algorithm for the studded system starts with

- The code starts by accepting four input arguments: u1, u2, u3, u4.
- Based on the inputs u1 and u2, the transfer function G(s) is computed and stored in the variable G.
- The value of parameter al is determined using u3, G, and u4.
- Initialization of various variables essential for the optimization process takes place. This includes setting the lower bound (lb) and upper bound (ub), as well as defining optimization parameters (beta, delta, max_iter) and population variables (x, s, fitness).
- The fitness of each population member is evaluated using an Objective Function
- The population member with the highest fitness value is identified as the best individual.
- The optimization process commences with a loop that runs for max_iter iterations.
- During each iteration, the position and scale of each population member are updated using random calculations.
- The position is perturbed and adjusted to be within the limits specified by lb and ub.
- The fitness of each population member is evaluated once again.
- If a fitter member is found, the best population member is updated accordingly.
- After the loop concludes, the optimized values of kp and kv are extracted from the best member and stored in the variables kp and kv, respectively.
- Finally, the function returns the values of kp and kv as the output.

## 3. SCO+BE for position control of armature controlled DC motor

### 3.1 Dynamic model of the studied DC motor

**Fig 6:**
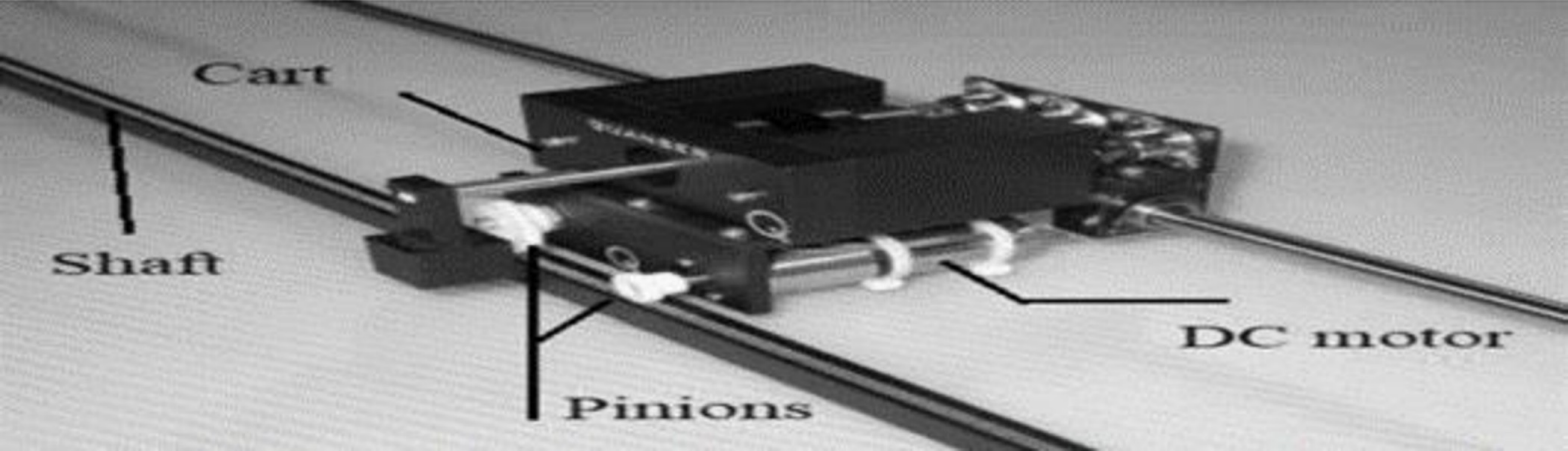
Studied cart system.

In this study, the focus is on a cart system that is propelled by a DC motor controlled by an armature. Fig 6 provides an illustration of this system. The cart itself is made of aluminum and is able to move along a shaft with a linear bearing. The movement of the cart is achieved through a track mechanism with a pinion, powered by a DC motor connected to a planetary gearbox [31,32].

The car’s system’s behavior may be represented by an open loop transfer function, which is as follows:

$$G(S)=\frac{X(S)}{Vm(s)}$$ (6)

with reference to the Newton’s second law:

$$M(\frac{d^{2}}{dt^{2}}x(t))+F_{ai}(t)=F_{c}(t)-B_{eq}(\frac{d}{dt}x(t))$$ (7)

to provide the armature inertial torque by:

$$F_{ai}=\frac{\eta_{g}K_{g}T_{ai}}{r_{mp}}$$ (8)

By utilizing Newton’s second law, one can determine that:

$$J_{m}(\frac{d^{2}}{dt^{2}}\theta_{m}(t))=T_{ai}(t)$$ (9)

The mechanical arrangement of the car’s rack and pinion system can be calculated as:

$$\theta_{m}=\frac{K_{g}X}{r_{mp}}$$ (10)

The motor’s driving force *F*_*C*_ can be calculated using:

$$F_{c}=\frac{\eta_{g}K_{g}T_{m}}{r_{mp}}$$ (11)

The DC motor’s torque can be stated as follows:

$$T_{m}=\eta_{m}K_{t}I_{m}$$ (12)

Furthermore, the angular velocity of the motor may be written as:

$$\omega_{m}=\frac{K_{g}(\frac{d}{dt}X(t))}{r_{mp}}$$ (13)

**Fig 7:**
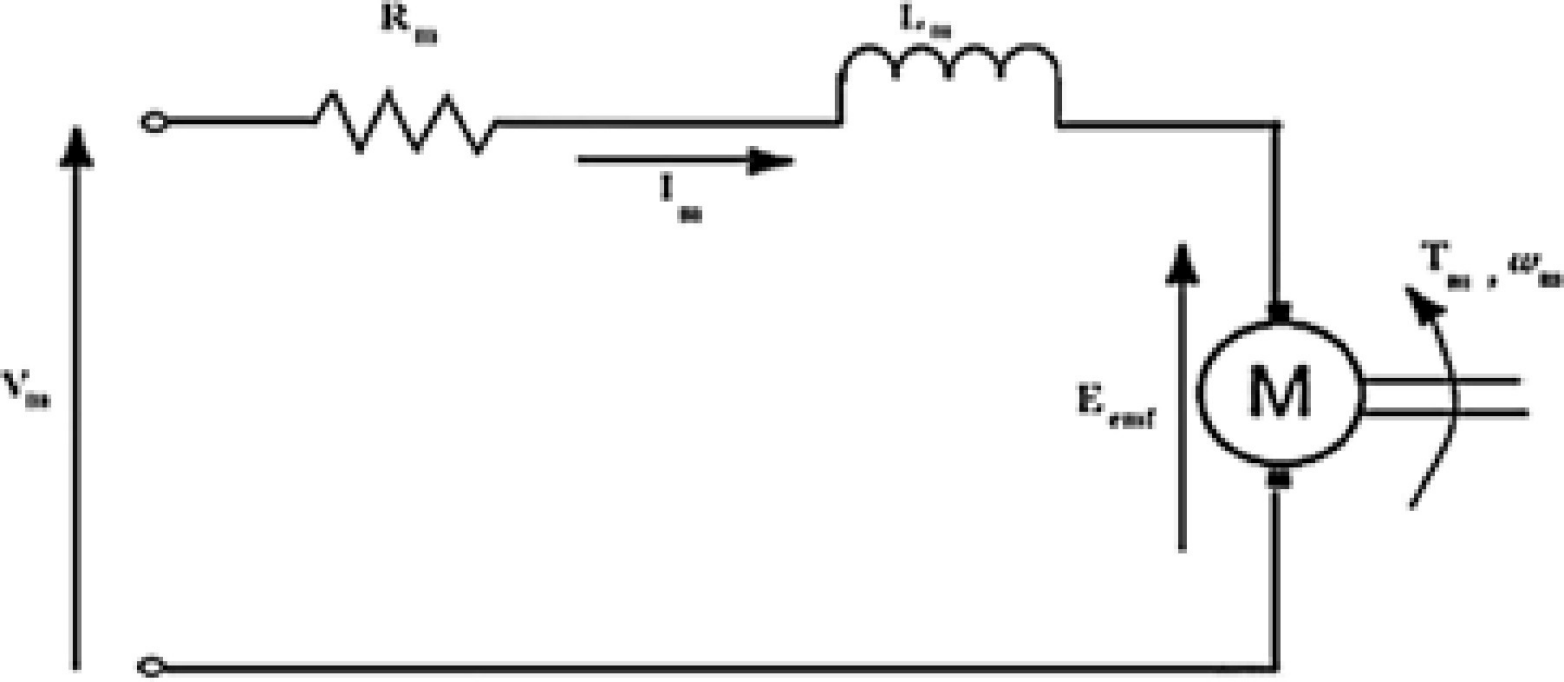
The DC motor’s armature circuit [1].

Fig 7 depicts the armature circuit of a typical DC motor. This electrical circuit’s use of Kirchhoff’s voltage law is illustrated as:

$$V_{m}-R_{m}I_{m}-L_{m}(\frac{d}{dt}I_{m})-E_{emf}=0$$ (14)

By ignoring the motor’s inductance, we get *I*_m_

$$I_{m}=\frac{V_{m}-E_{emf}}{R_{m}}$$ (15)

Finally, the motor’s (TF) can be determined as:

$$G(S)=\frac{r_{mp}\eta_{g}K_{g}\eta_{m}K_{t}}{(R_{m}Mr_{mp}^{2}+R_{m}\eta_{g}K_{g}^{2}J_{m})S^{2}+(\eta_{g}K_{g}^{2}\eta_{m}K_{t}K_{m}+B_{eq}R_{m}r_{mp}^{2})S}$$ (16)

**Fig 8:**
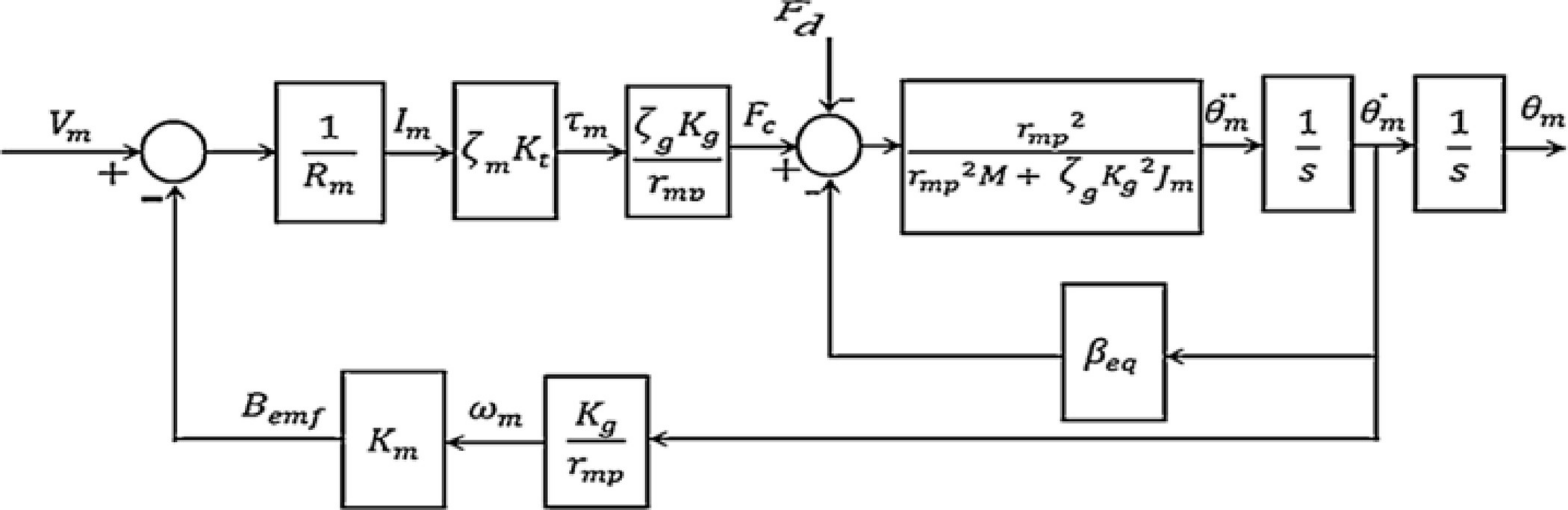
Block diagram illustrating the design of the used DC motor.

The block diagram for the under-study armature-controlled DC motor is shown in Fig 8, which gives detailed information. Table 1 [31] contains thorough information on the system’s elements. Table 2 also shows the maximum values related to the electrical motor.

**Table 1:** Studded Values of system parameters.

Symbol	value	Symbol	value
*ζ*_*g*_	100%	*r*_*mp*_	6.35*10^−3^ M
*ζ*_*m*_	100%	*K*_*g*_	3.71
*K*_*t*_	7.67*10^−3^ N.m/A	*R*_*m*_	2.6 ohm
*M*	0.97 Kg	*J*_*m*_	3.9*10^−7^ Kg. *m*^2^

**Table 2:** Studded electrical motor parameters maximum values.

Symbol	value	Symbol	value
*V*	6 V	*I*	1 A
*F*	50 HZ	Ω	628.3 rad/sec

### 3.2 Controlling of the studded system using SCO-based adaptive position control

#### 3.2.1 Standard adaptive PV controller based on SCO

**Fig 9:**
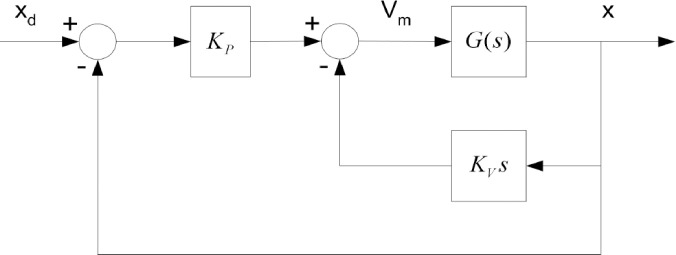
Basic PV controller design.

The design of the PV controller is seen in Fig 9. The model of the previously mentioned armature-controlled DC motor is represented by *G*(*S*) in this diagram. The parameter *K*_*p*0_, which is the nominal value of *K*_*p*_, and the parameter *K*_*v*0_, which is the nominal value of *K*_*v*_, can be illustrated as follows:

$$\begin{array}{l} K_{p0}=274.62\;v/m\\ K_{v0}=5.532\;v.sec/m \end{array}$$

In order to attain system characteristics of 10% overshoot and a rise time of 0.15 sec, these specific values of *K*_*p*0_ and *K*_*v*0_ were used.

The objective of SCO optimization is to optimize the gains Kp and Kv, of the PV controller.

**Fig 10:**
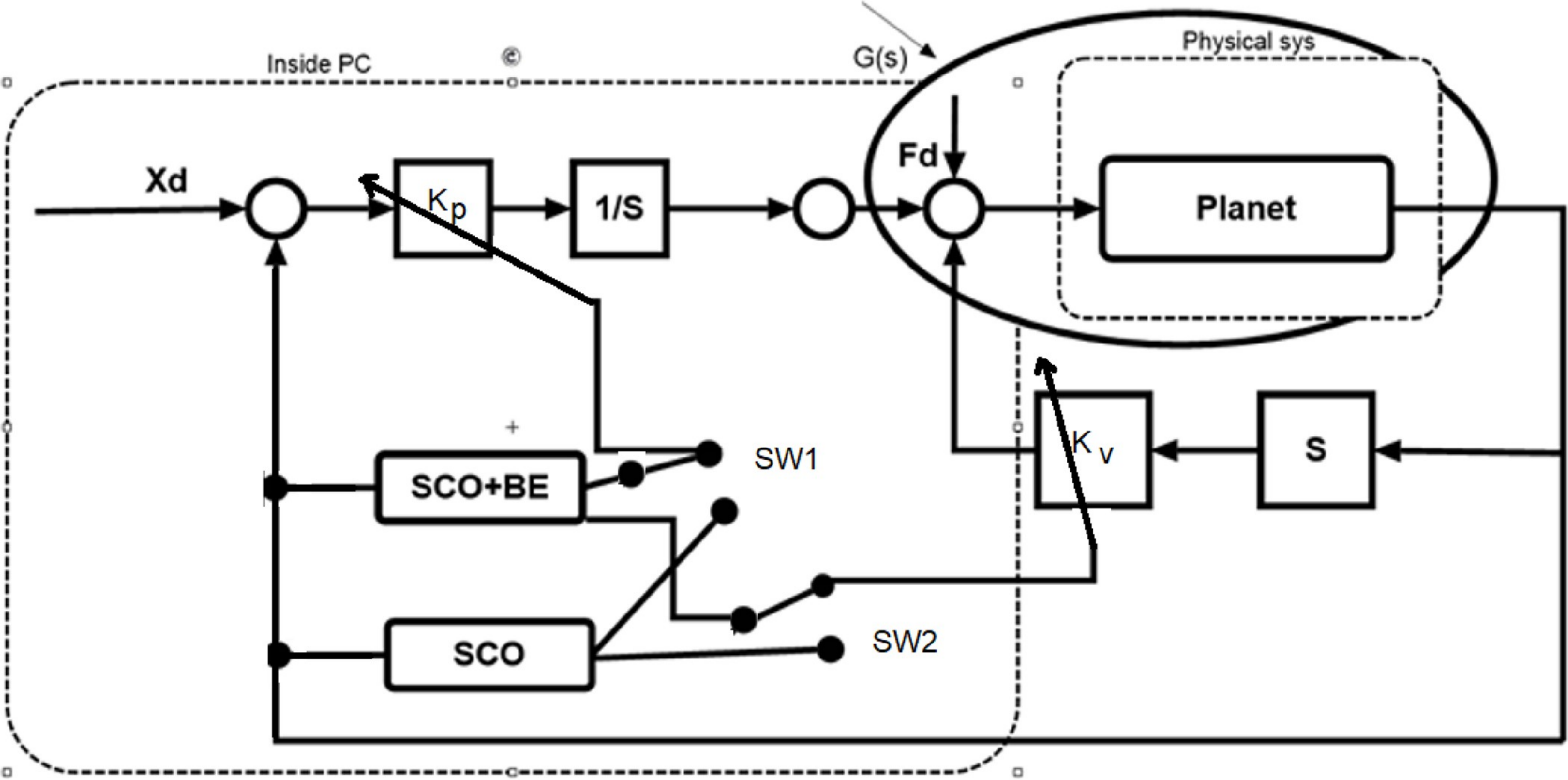
Block schematic of the controller-equipped system.

Fig 10 illustrates the implementation of a DC motor’s adaptive position controller using the classical SCO optimization method. To optimize the SCO objective function (OF), it is necessary to calculate the closed loop transfer function of the system using Eq (16) and taking into account the nominal data provided in Table 1 then

$$G_{o}(S)=\frac{2.46}{s^{2}+17.13s}$$ (17)

Additionally, as illustrated in Fig 10 the feedback transfer function may be stated as:

$$\frac{X_{i}}{X_{di}}=\frac{2.46^{*}K_{pi}}{S^{2}+(17.13+2.46^{*}K_{vi})S+(2.46^{*}K_{pi})}$$ (18)

By observing Eq (18), we can observe that during any iterations, the variables *ω*_*ni*_,*η*_*i*_,*T*_*ri*_,*T*_*si*_,*M*_*pi*_, and *J*_min_ are solely dependent on the modified gain values of the PV controller, *K*_*pi*_ and *K*_*vi*_,.

#### 3.2.2 Modified adaptive PV controller based on SCO+BE

The system closed loop transfer function can be calculated during any iteration using Eqs (5) and (16), and the structure depicted in Fig 8, as demonstrated in Fig 10 which shows the position control system using the proposed SCO with BE.


$$\frac{X_{i}}{X_{di}}=\frac{2.46^{*}(\prod_{n=1}^{i}AL_{n})*K_{pi}}{S^{2}+(17.13+2.46^{*}{K_{vi}}^{*}(\prod_{n=1}^{i}AL_{n}))S+(2.46^{*}{K_{pi}}^{*}(\prod_{n=1}^{i}AL_{n}))}$$ (19)

Where

$$\begin{array}{c} \omega_{ni}=\sqrt{2.46*(\prod_{n=1}^{i}AL_{n})K_{pi}} \end{array}$$

And

$$\begin{array}{c} \eta_{i}=\frac{\left(17.13+2.46*K_{vi}^{*}(\prod_{n=1}^{i}AL_{n})\right)}{2*\omega_{ni}} \end{array}$$

The values of *ω*_*ni*_ and *η*_*i*_ are used to calculate the parameters *M*_*pi*_,*T*_*ri*_ and *T*_*si*_ for the closed-loop system of second order. It is clear that the minimum objective function in *J*_min_ SCO is influenced by the values of *K*_*pi*_, *K*_*vi*_, and the term $\left(\prod_{n=1}^{l}AL_{n}\right)$, which represents system disturbance and parameter variations.

How the problem and algorithm interact is demonstrated in the following scenario: During each iteration, while the computer executing the code of SCO and BE it receives the plant’s input *U*_*i*_ and the output *X*_*i*_. The values of these signals will be used to compute *G*_*i*_(*S*) using Eq (2), and the previously stored value of *G*_*i*−1_(*s*) will be used to determine the value of *AL*_*i*_. The value of ($\prod_{n=1}^{l}AL_{n}$) will be determined using this value and its previous cumulative values that were previously saved. Meanwhile, the loops of SCO algorithm will generate potential values of *K*_*pi*_, *K*_*vi*_ which will be used with $\left(\prod_{n=1}^{l}AL_{n}\right)$ to calculate the parameters of the time response *M*_*pi*_,*T*_*ri*_ and *T*_*si*_. These parameters will be used to determine the value of the objective function *J*_min *i*_, which eventually determines the ideal and final values of *K*_*pi*_, *K*_*vi*_. These values will be provided to the PV controller’s Simulink program, which will produce the control signals and transmit them to the physical process via a Data Acquisition card. The suggested adaptive control scheme’s effectiveness is influenced by the computer’s technical parameters, including its RAM, cache memory, and CPU speed.

### 3.3 Simulation results for proposed controller of DC motor application

The Simulink package of MATLAB used as a simulation environment for the DC motor (with parameters listed in Table 1) system with the proposed control algorithm, in addition, the parameters of the suggested SCO is illustrated in Table 3.

**Table 3:** Parameters of SCO.

bounds of K_p0_	[265:280]
bounds of K_v0_	[4:6.5]
max_iter = 100	maximum number of iterations
beta = 0.5	Constant for updating scales
Delta = 0.1	Perturbation factor

The modified SCO approach includes the following variables.

The chosen object function has been utilized.


$$J_{\min}=\sum (M_{p}^{2}+T_{r}^{2}+T_{s}^{2})$$

**Fig 11:**
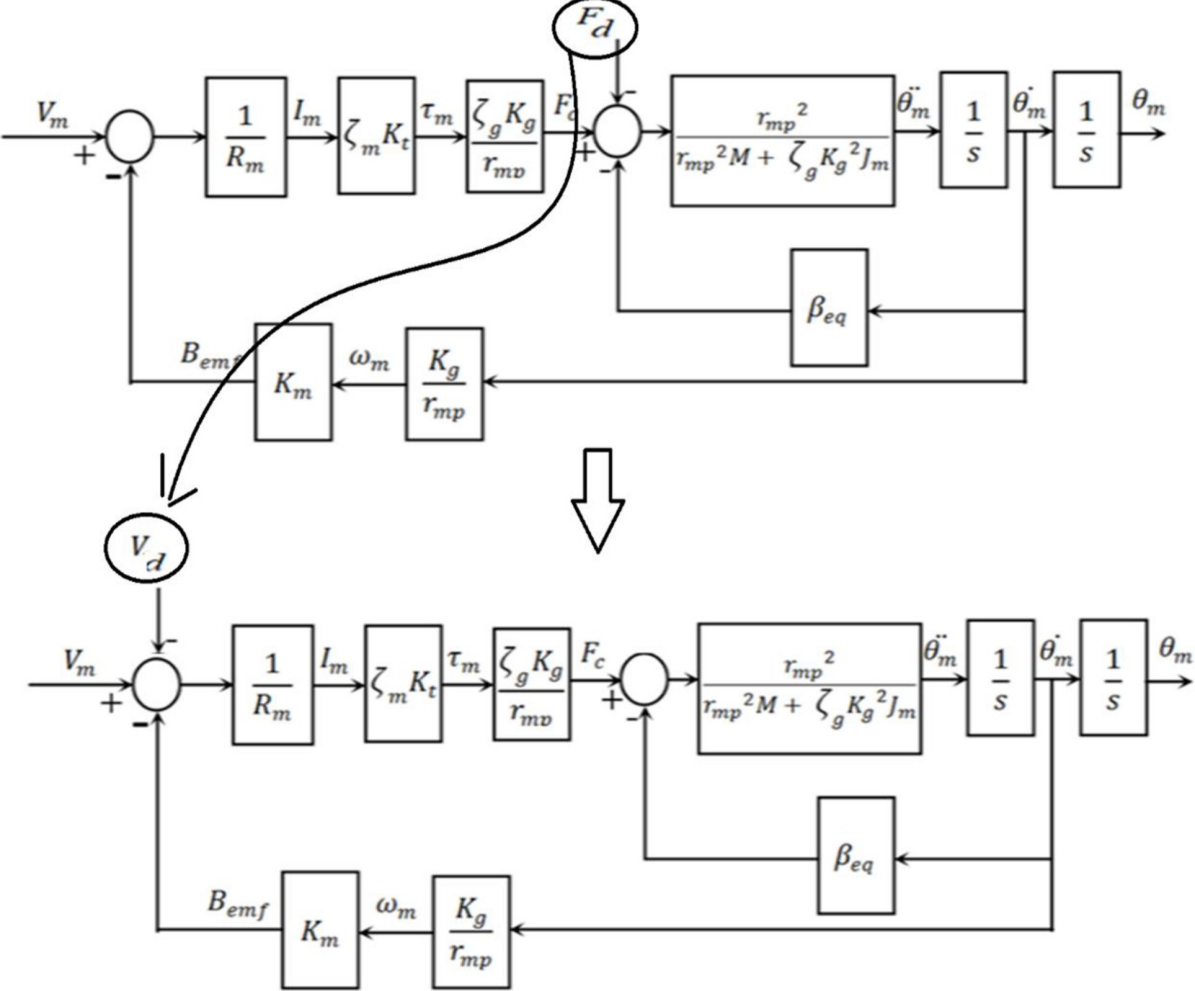
The option of sum point movement.

**Fig 12:**
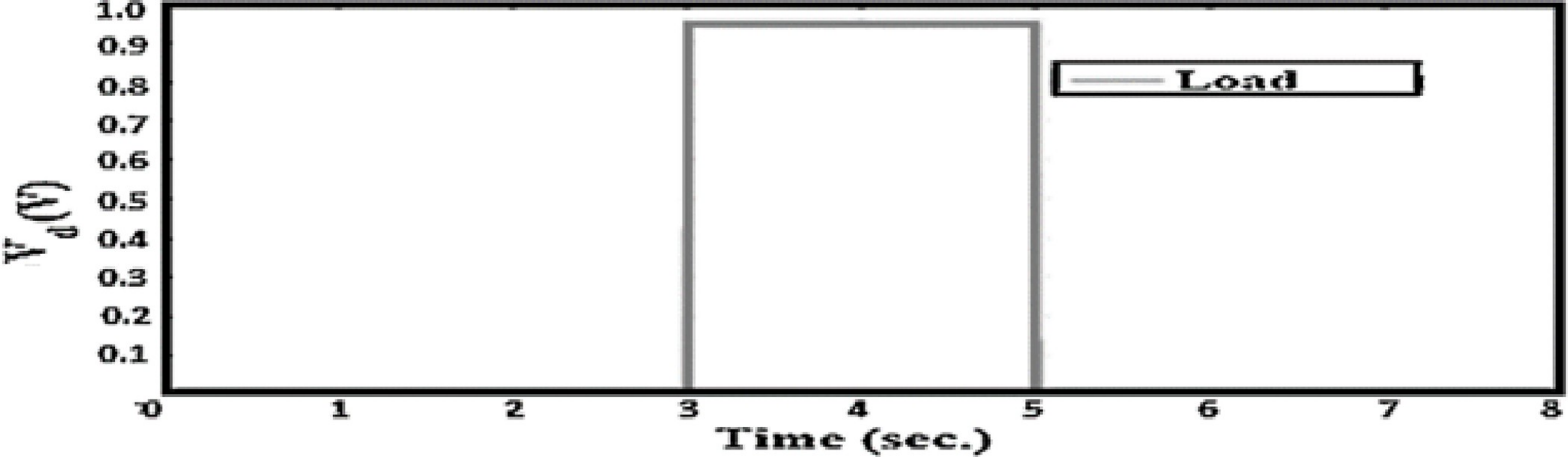
Load disturbance.

As shown in Fig 11, the physical disturbance has been replaced with an equivalent voltage using the sum point movement option to make it measurable. The system under analysis has been tested for two scenarios: a sudden change in desired input and a sudden load disturbance. To observe the impact on the proposed system, an equivalent load disturbance has been introduced from t = 3 seconds to t = 5 seconds. The value of the load disturbance is 0.95 volts, as shown in Fig 12. Furthermore, the desired motor position signal starts at 1.5 seconds with a magnitude of 15 mm and ends at 6 seconds.

**Fig 13:**
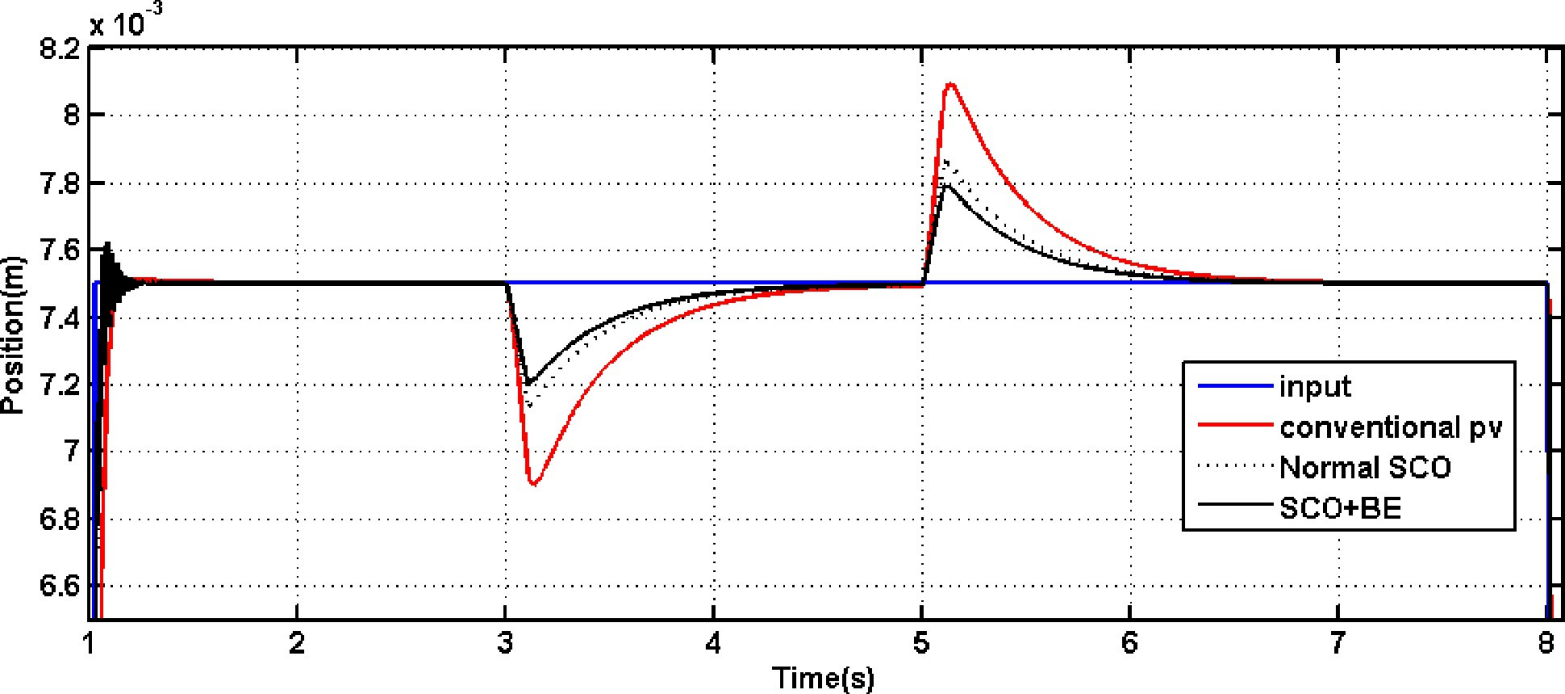
The outcome of tunning PV controller using conventional SCO/SCO+BE in the event of a load disturbance.

**Fig 14:**
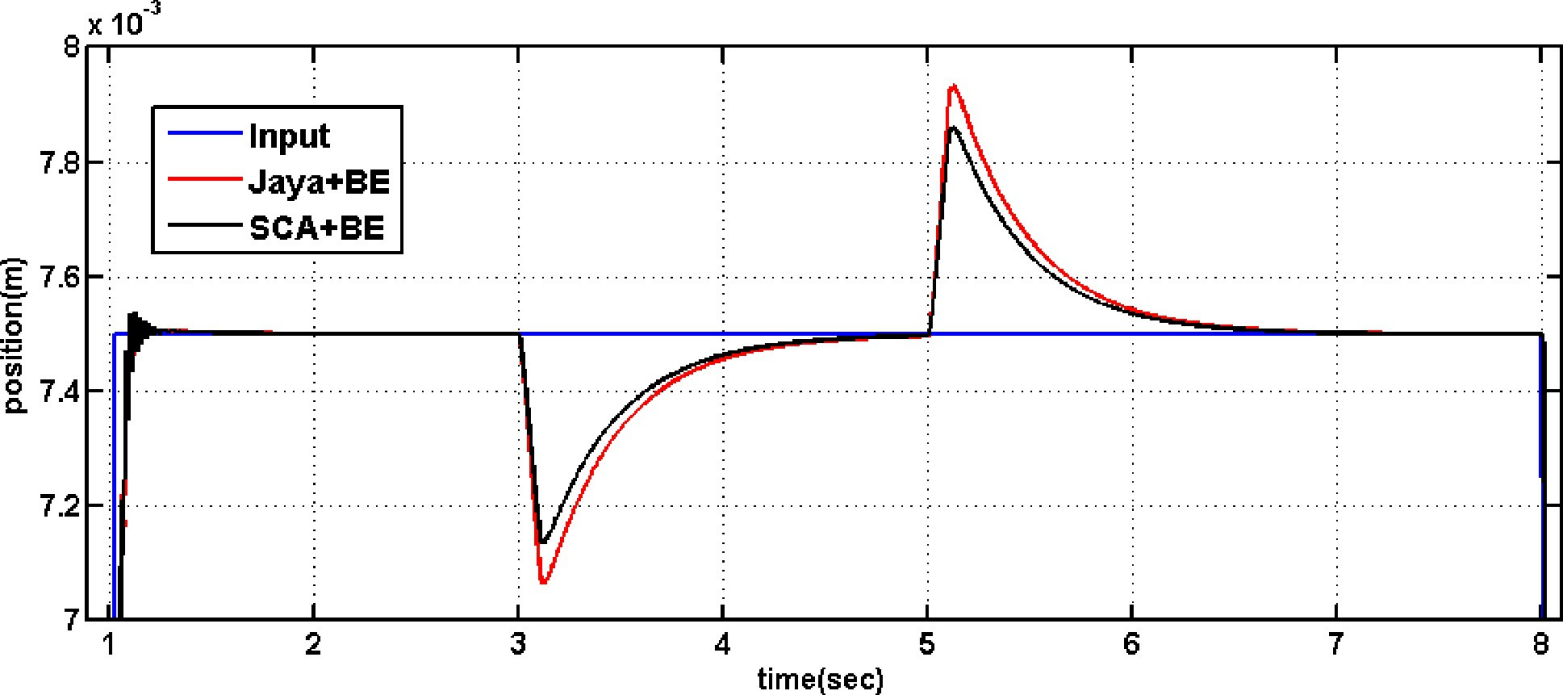
The outcome of tunning PV controller using conventional Jaya+BE /SCO+BE in the event of a load disturbance.

Fig 13 displays the outcomes of comparing the performance of systems employing the conventional PV controller, Controller tuned by traditional SCO and that one adapted using the modified SCO with BE during load disturbance and step reference change. It is evident from Fig 13 that the rise time with the proposed SCO + BE is about 0.08 sec, while it is about 0.2 sec with the normal SCO. The PV controller tuned by the proposed SCO + BE also improves the overshoot during step disturbance. Also Fig 13 demonstrates that the suggested PV controller tuned by normal SCO or modified SCO + BE successfully handles load disturbance. Furthermore, the performance with the modified SCO technique is superior to that of the normal SCO. also, comparison has been made between SCO+BE and Jaya+BE (presented in [32]) in Fig 14 leads to an improvement of the overshoot during step disturbance of the proposed SCO + BE than jaya algorithm. Detailed parameters can be found in Table 4. From Table 4, it can be observed that the proposed SCO + BE yields the best overshoot, rise time, and settling time compared to other controllers. Both Figs 13 and 14 demonstrate that the suggested PV controller tuned by modified SCO + BE successfully handles load disturbance. Furthermore, the performance with the SCO+BE technique is superior to that of the normal SCO and jaya algorithm.

**Table 4:** Time response parameters.

	*M*_*p*_	*T*_*r*_(sec)	*T*_*s*_(sec)
Conventional PV	2.3%	0.2	0.5
Normal SCO	1.5%	0.2	0.5
jaya+BE	1.4%	0.1	0.47
SCO+BE	1.3%	0.08	0.35

### 3.4 Experimental results for proposed controller of DC motor application

**Fig 15:**
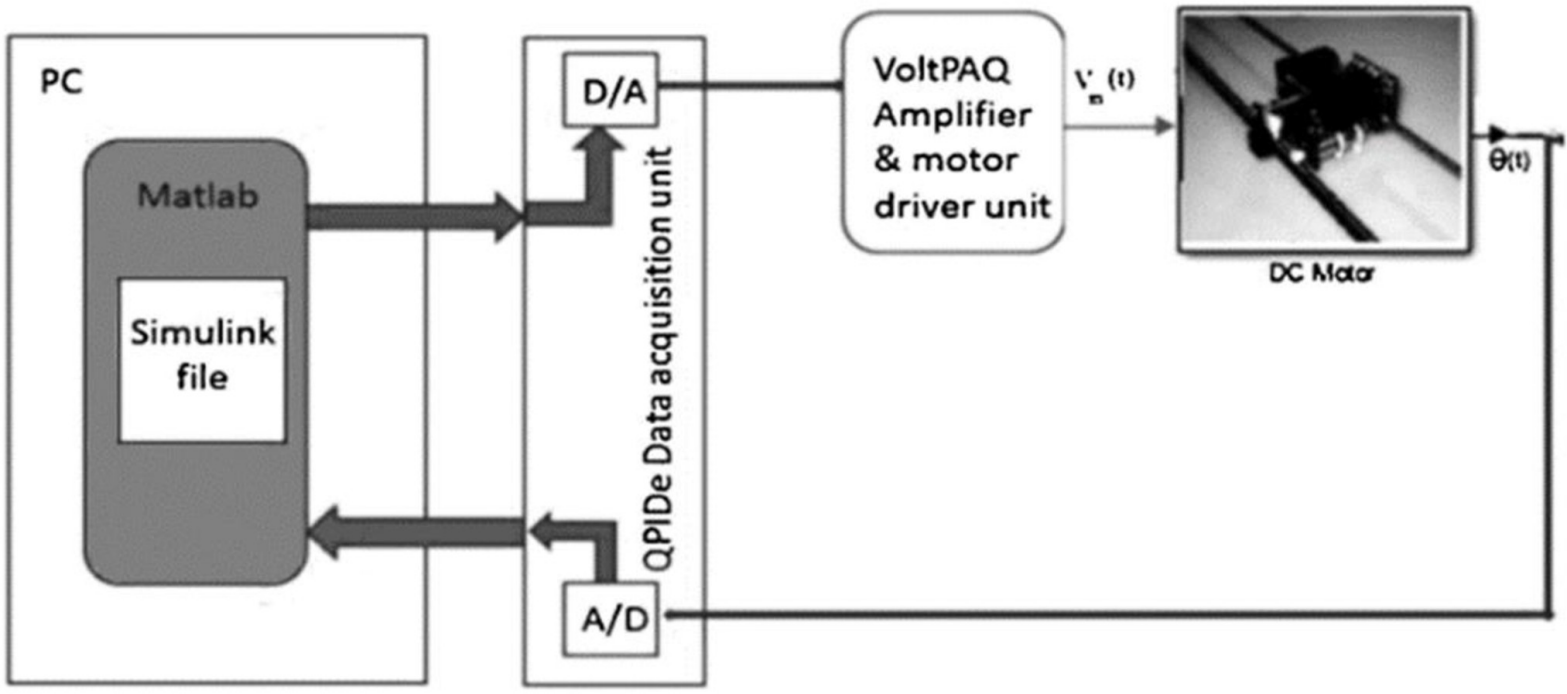
The proposed system using digital MATLAB controller.

**Fig 16:**
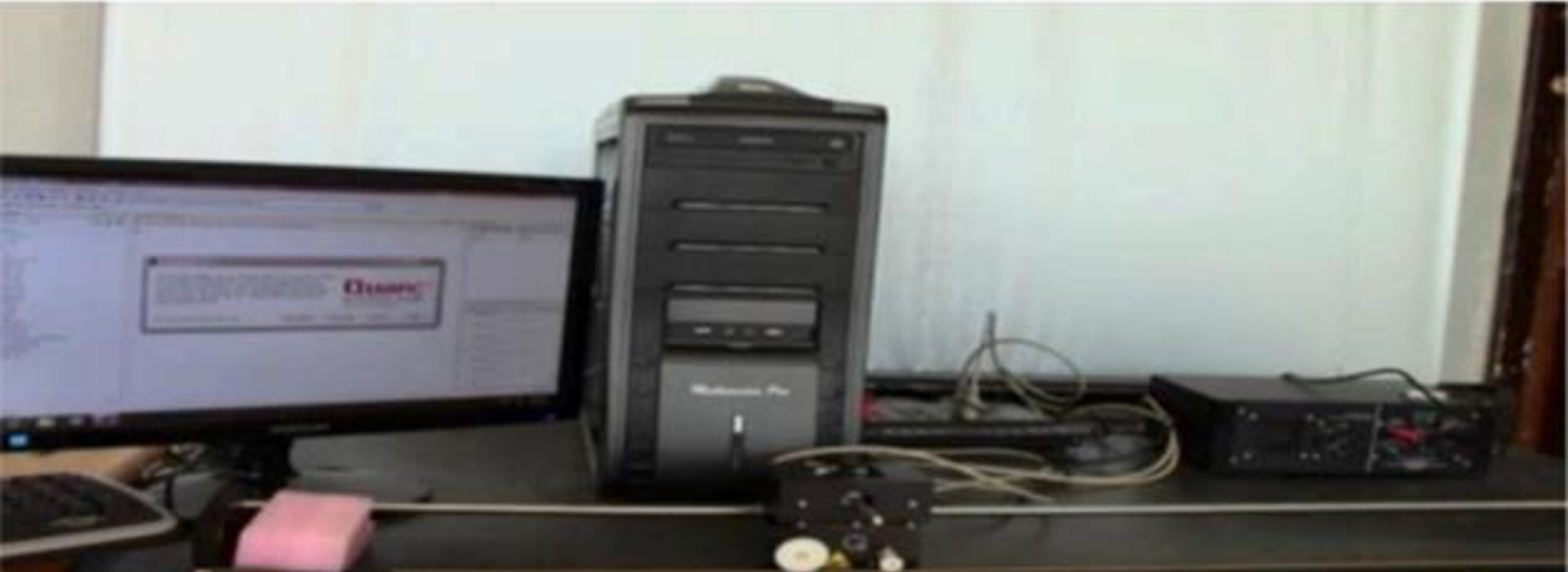
Experimental setup.

**Fig 17:**
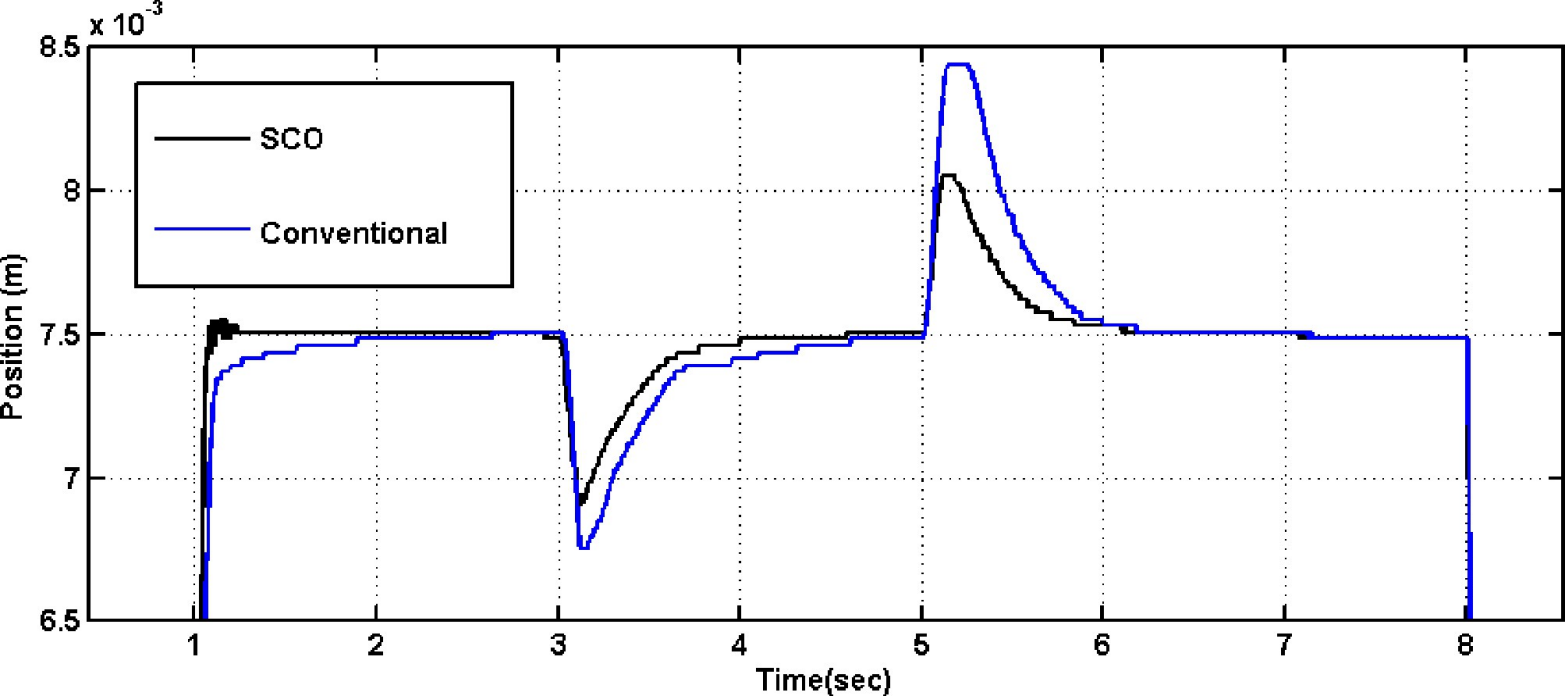
The outcome of tunning PV controller using conventional SCO in the event of a load disturbance.

**Fig 18:**
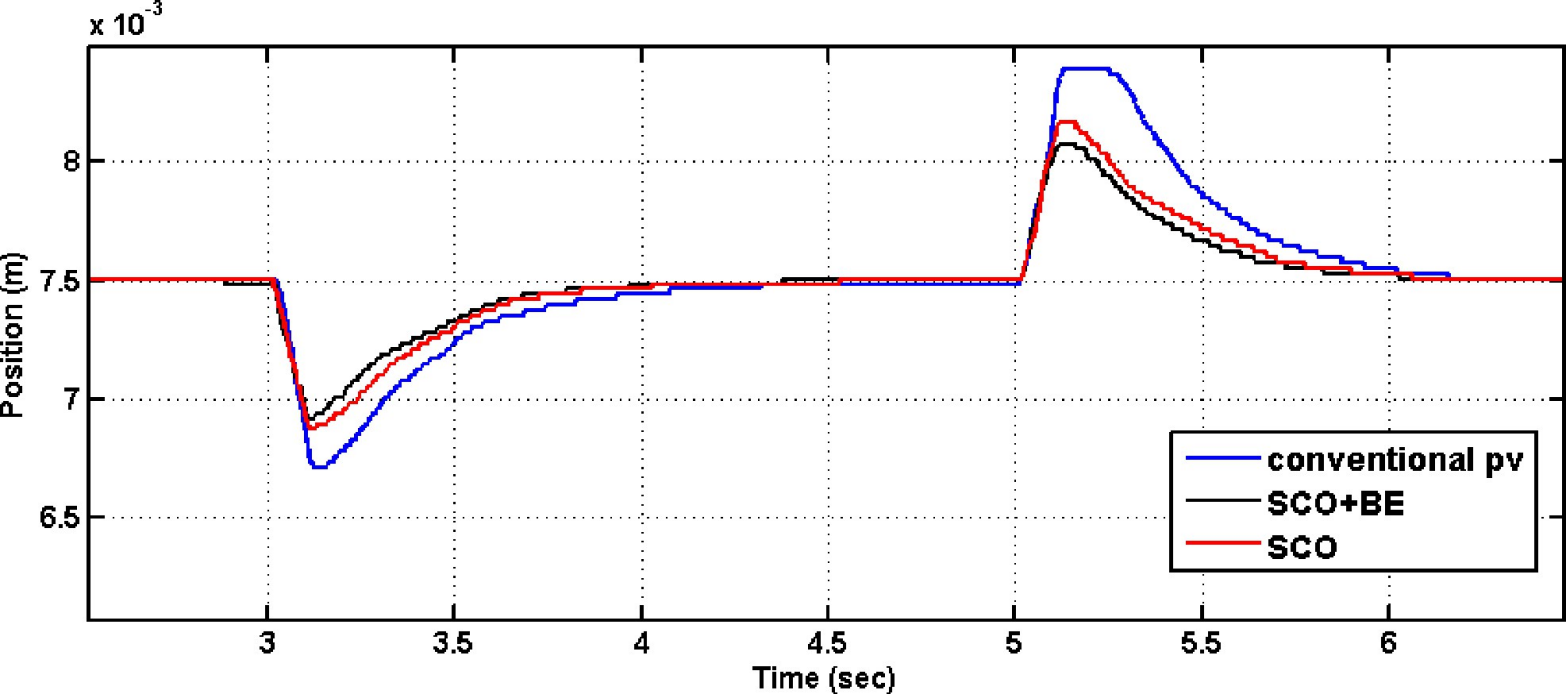
The outcome of tunning PV controller using conventional SCO/SCO+BE in the event of a load disturbance.

The experimental implementation of the suggested car powered by a DC motor is seen in Fig 15. QPIDe data acquisition card and QuanserVoltPAQ were used to communicate between MATLAB executed in the PC and the DC motor. A single-ended optical shaft encoder was act as a position sensor. Fig 16 shows how the hardware is organized. The identical circumstances that were utilized in simulation testing were reproduced experimentally. as shown in Figs 17 and 18. Fig 17 demonstrates the system responses for classical and adaptive PV controllers. It was found that the suggested technique had a positive impact on the system response in the face of step load change and step input. Comparing the value acquired using the typical PV controller to the value obtained using standard SCO, the overshoot dropped to roughly 35%.

Additionally, the results shown in Fig 18 demonstrate that the system utilizing the modified SCO+BE outperforms the system utilizing the normal SCO algorithm in the presence of load disturbance. It is evident that the system response achieved using the modified SCO+BE is smooth and stable, while the response obtained using the standard SCO algorithm is unfavorable in the initial three seconds, characterized by high ripples. according to the experimental data, the system using the PV controller tuned by the suggested SCO+BE technique is clearly more resilient and robust than the system using the controller optimized by the traditional SCO method. Furthermore, a comparison between SCO and SCO+BE is conducted at selected time instances (t = 3 sec. and t = 5.5 sec.), and the obtained data is recorded in Table 5 using the program code. The data presented in Table 5 confirms that during critical moments such as the introduction and removal of step load disturbance, the system with the controller tuned by SCO+BE exhibits favorable values for T_r_, T_s_, M_p_ and ISE compared to the system with the controller tuned by standard SCO.

**Table 5:** An optimization comparison between SCO and SCO + BE.

	SCO						SCO+BE					
T(sec)	*K*_*pi*_	*K*_*vi*_	|*M*_*pi*_|	*T*_*ri*_	*T*_*si*_	ISE	*K*_*pi*_	*K*_*vi*_	|*M*_*pi*_|	*T*_*ri*_	*T*_*si*_	ISE
3	910	44	1.9%	0.21	0.503	1.4	780	32	1.03%	0.03	0.5	0.50
5.5	738	47	1.6%	0.19	0.5	0.74	615	38	0.99%	0.95	0.053	0.36

To support the past simulation and experimental extracts, some statistical analysis have been done. Performance metrics, including mean square error (MSE), maximum error (Max E), minimum error (Min E), and standard deviation (STD E), were selected to assess the accuracy of position control. Each method underwent three trials, and the MSE, Max E, Min E, and STD E values were computed for each trial. The resulting metrics for the trials are as shown in Table 6.

**Table 6:** Statistical analysis for I, SCO and SCO+BE.

	Conventional controller	Normal SCO	SCO+BE	
	0.032	0.025	0.015	**MSE**
**TRAIL 1**	-0.08	-0.06	-0.04	**Min error**
	0.1	0.08	0.05	**Max error**
	0.015	0.012	0.008	**STD E**
	0.033	0.023	0.012	**MSE**
	-0.07	-0.04	-0.02	**Min error**
**TRAIL 2**	0.09	0.05	0.03	**Max error**
	0.014	0.01	0.006	**STD E**
	0.035	0.024	0.011	**MSE**
	-0.09	-0.05	-0.01	**Min error**
**TRAIL 3**	0.1	0.07	0.02	**Max error**
	0.016	0.011	0.005	**STD E**

Regarding the analysis of position control accuracy for the armature-controlled DC motor, the mean square error (MSE) values for three different methods, namely SCO+BE, normal SCO, and the conventional controller were found to be 0.0126, 0.024, and 0.033, respectively. Comparing the maximum error (Max E), SCO+BE exhibited a value of 0.03, while normal SCO and the conventional controller had values of 0.08 and 0.1, respectively. Lower (Max E) values indicate superior tracking precision. In terms of minimum error (Min E), SCO+BE yielded values of -0.04, while normal SCO and the conventional controller had values of -0.06 and -0.08, respectively. Smaller (Min E) values imply better positional accuracy. Analyzing the standard deviation (STD E), SCO+BE showed a value of 0.006, whereas normal SCO and the conventional controller had values of 0.01 and 0.014, respectively. Smaller (STD E) values indicate more consistent control performance. In conclusion, the statistical analysis clearly demonstrates that the proposed SCO+BE algorithm outperforms both normal SCO and the conventional controller in terms of position control accuracy. SCO+BE consistently shows lower MSE, Max E, Min E, and STD E values, indicating improved tracking precision, positional accuracy, and control consistency. These results validate the effectiveness and superiority of the SCO+BE algorithm for adaptive position control of the cart, surpassing traditional methods.

## 4. SCO+BE for LFC of isolated microgrid

### 4.1 Dynamic Model of an isolated microgrid

**Fig 19:**
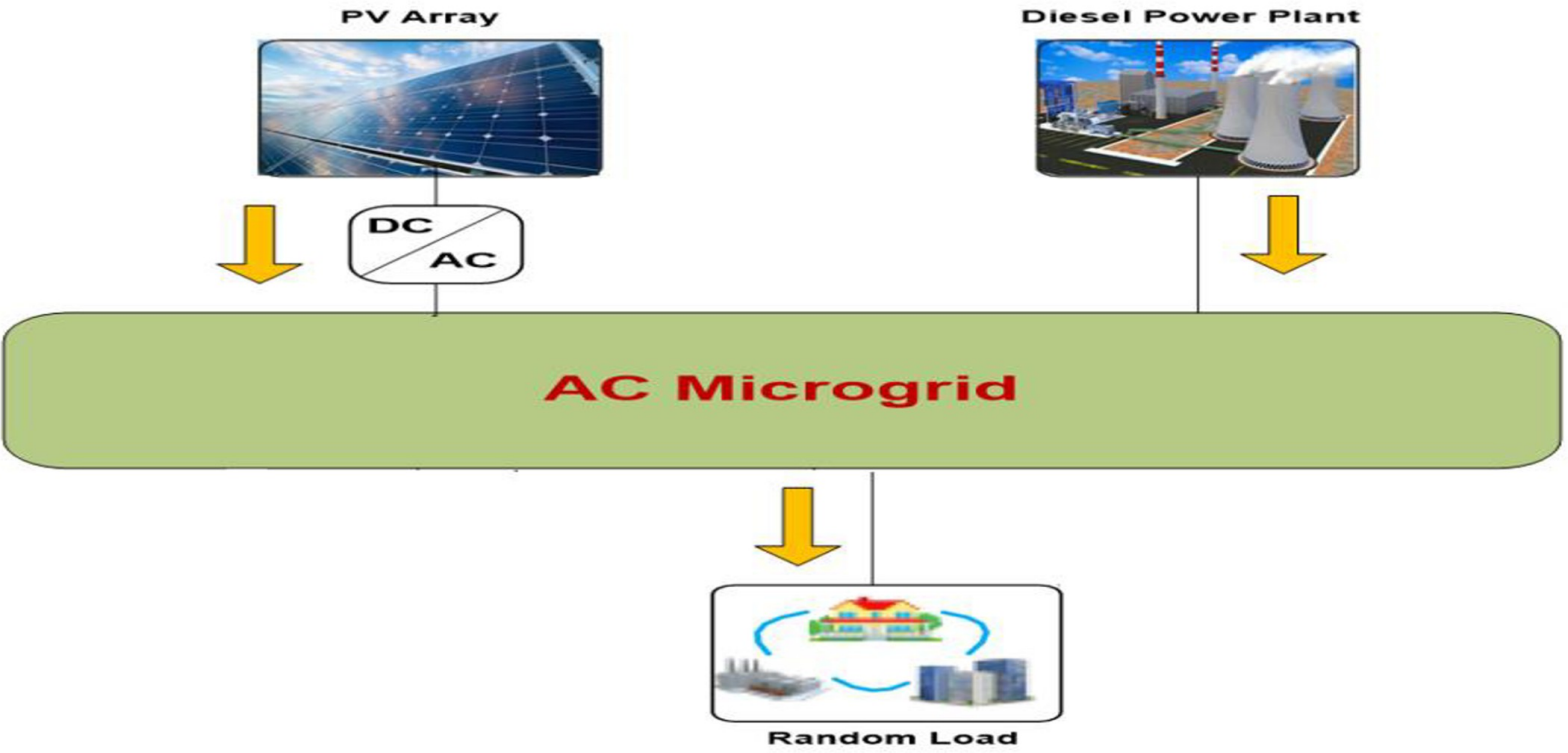
Power flow chart of the studied microgrid.

**Fig 20:**
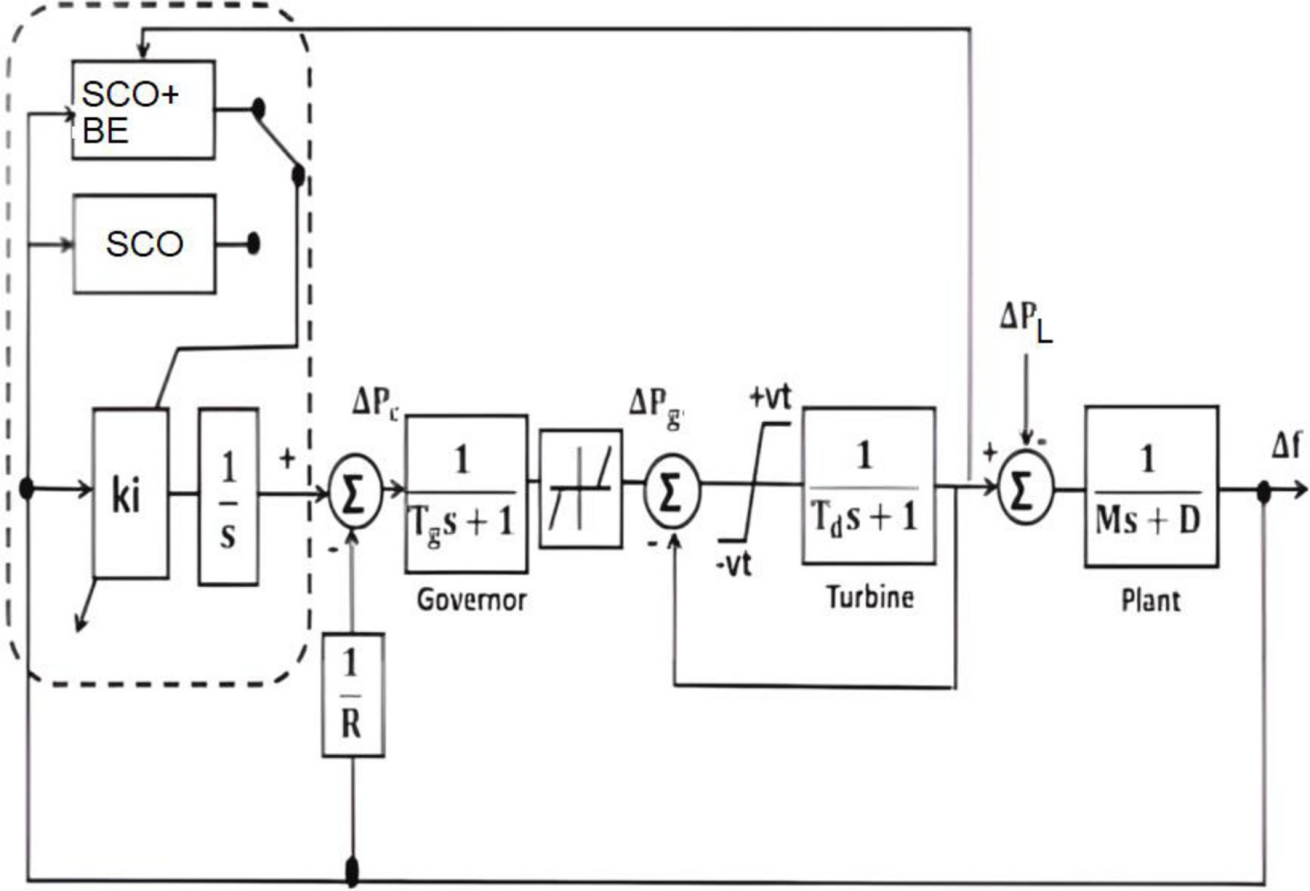
Block diagram of the model of microgrid power system.

Applying the suggested control method to other application is an effective method to evaluate it. A chosen application is an isolated microgrid power system. Fig 19 illustrates the power flow graph of the proposed microgrid while Fig 20 shows the block diagram of a chosen micro-grid power system. Eqs 20–22 can be used to characterize the dynamic model of the suggested micro-grid power system. The supply error (ΔPd—ΔPL) and frequency variation (Δf) have a dynamic connection that may be represented as the total load-generator;:

$$\Delta f=(\frac{1}{M}).\Delta Pd-(\frac{1}{M}).-\Delta PL-(\frac{D}{M}).\Delta f$$ (20)

The dynamic of the diesel generator may be written as:

$$\Delta Pd=(\frac{1}{Td}).\Delta Pg-(\frac{1}{Td}).\Delta Pd$$ (21)

The dynamic of the governor may be written as:

$$\Delta Pg=(\frac{1}{Tg}).\Delta Pc-(\frac{1}{R.Td}).\Delta f-(\frac{1}{Tg}).\Delta Pg$$ (22)

The parameters of the studied microgrid system is illustrated in Table 7.

**Table 7:** Parameters of the studied micro-grid.

D	H = (M/2)	R	Tg	T_d_
**(Pu/Hz)**	**(Pu.sec)**	**(Hz/Pu)**	**(sec)**	**(sec)**
0.015	0.08335	3	0.08	0.4

### 4.2 Adaptive LFC based SCO+BE

**Fig 21:**
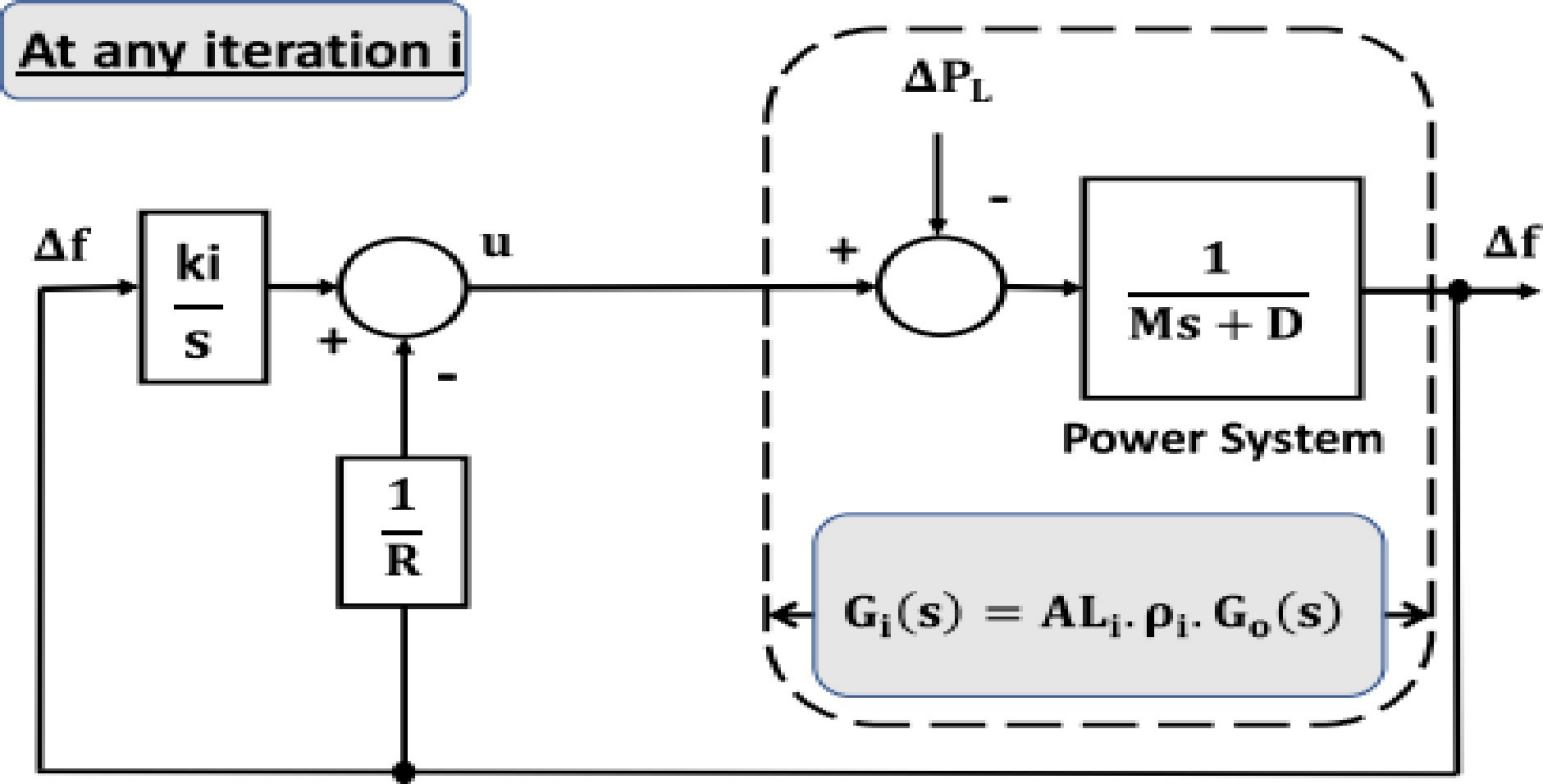
Reduced model of the studied microgrid.

Fig 21 illustrates a simplified μG model; it is employed to determine the restricted area’s FO closed-loop system’s variables.

$$T.F=\frac{{wn}^{2}}{S^{2}+2\eta Wn+{Wn}^{2}}=\frac{\frac{Ki}{Mo}}{S^{2}+(\frac{\left(Do+\frac{1}{Ro}\right)}{Mo})S+\frac{Ki}{Mo}}$$ (23)

where *D*_*o*_, *R*_*o*,_ and *M*_*o*_ are the nominal values of *D*, *R*, and *M*, respectively

$$\omega_{n}=\sqrt{Ki/Mo},\;\eta =\frac{\frac{\left(Do+\frac{1}{Ro}\right)}{Mo}}{2\omega_{n}}$$

$$T_{r}=\frac{\pi -\sqrt{\left(1-\eta^{2}\right)}}{\omega_{n}\sqrt{\left(1-\eta^{2}\right)}},\;T_{s}=\frac{4}{{\eta \omega }_{n}},M_{P}=e^{\frac{-\pi \eta }{\sqrt{\left(1-\eta^{2}\right)}}}$$

This is the OF of the SCO-based BE identifier.


$$J=min\sum {(T}_{r}+T_{s}+M_{P})$$

This means that the OF (*J*) is a function of *AL*_*i*_ and *k*_*i*_ to address the system challenges.

### 4.3 Simulation results of microgrid LFC based SCO+BE

**Fig 22:**
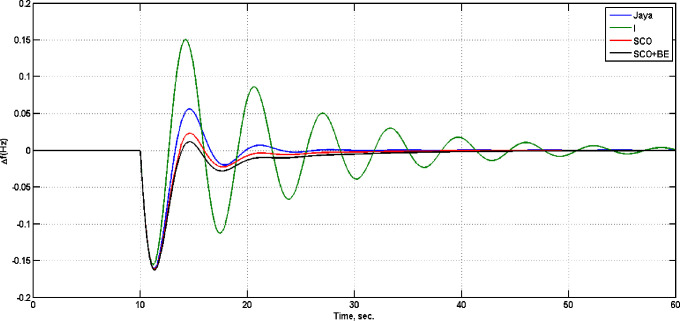
Frequency deviation.

**Fig 23:**
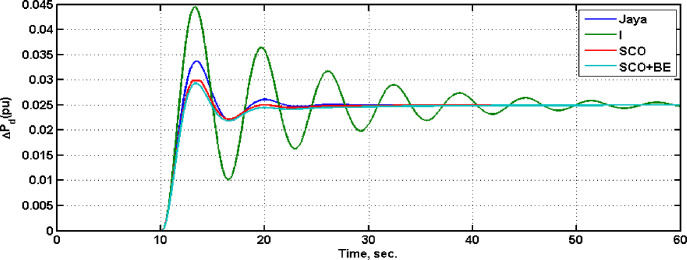
Diesel power deviation.

To evaluate the effectiveness of the proposed adaptive control algorithm, system with SCO+BE has been compared with that one with only SCO with parameters shown in Table 8, Jaya(with detailed listed in [32]) and classical I in case of step load change from 0 to 0.025 pu at t = 10, Fig 22 illustrate the frequency responses of the four controllers at this case, while Fig 23 shows the diesel power deviations at the same situation. From Figs 22 and 23 its clear that system with SCO+BE is superior from the time response viewpoint.

**Table 8:** Data of SCO.

Maximum Iteration (IT max)	100
Population Size (k)	5
Convergence constant (a)	2

## 5. Conclusion

This paper proposes an innovative adaptive controller that utilizes a modified SCO algorithm to individually adjust the gains of traditional controller parameters. In order to enhance the performance of the standard SCO a new modification method called the "Balloon Effect (BE)" is introduced. The BE approach incorporates both the values of the tuned gain parameters and the system changes, represented by the parameter ALi, into the objective function of the SCO algorithm This modification increases the algorithm’s sensitivity to load disturbances and uncertainties in the system parameters, resulting in improved system characteristics.

The proposed approach has been used to optimize a PV controller’s gains for controlling the linear position of a cart powered by a DC motor and to make online tuning of load frequency controller in isolated microgrid. By combining BE with SCO, Improvement has been made to the control method’s sensitivity to load disturbance and system parameter uncertainty for both systems. Additionally, incorporating BE into the SCO algorithm has led to improvements in the system’s general characteristics, including rise time, settling time, and over shoot. The proposed adaptive controller using SCO+BE has been evaluated against external step disturbances and has demonstrated excellent and distinguished results. Comparisons between the system with modified SCO, normal SCO and jaya optimization algorithm under external disturbances and system parameter changes have been made, with simulation and experimental results supporting the superiority of the proposed SCO+BE system in all study cases.

## 6. Future works

- Exploring the application of the proposed approach to diverse optimization techniques, including whale Search, Bat optimization algorithm, and ADAM optimization.
- Additionally, investigating the potential improvement of the proposed control algorithm in other industrial applications

## Supporting information

S1 File (ZIP)

## Abbreviations

Symbol: Variable
R: random value from 0 to 1
J: Number of designed variables
K: population size
I: Number of iterations
*X*(*S*): the position of the car
*V*_*m*_(*S*): motor voltage
I_m_: motor current
R_m_: electric resistance of the motor
L_m_: Inductance of the motor
E_emf_: back electromotive force voltage
K_t_: Motor torque constant
M: The total mass of the car system
F_ai_: armature rotational inertial force
F_C_: Car driving force produced by the motor
B_eq_: equivalent viscous damping coefficient
ζ_g_: efficiency of gearbox
r_mp_: motor pinion radius
K_g_: planetary gearbox ratio
T_ai_: armature inertial torque
J_m_: rotor moment of inertia
K_t_: motor torque constant
ζ_m_: Efficiency of the motor
V_m_: voltage motor
ΔPg: The variation in the governor’s output
ΔPd: Change in diesel power
Δf: Frequency deviation
ΔPL: Load deviation
ΔPc: additional control action
M: Equivalent inertia constant
D: Equivalent damping coefficient
R: Speed drop characteristic
Tg: Time constant of the governor
Td: Time constant of the Turbin
